# Liver metastasectomy-cytoreductive surgery- hyperthermic intraperitoneal chemotherapy and ileal pouch-anal anastomosis: A case report

**DOI:** 10.1016/j.ijscr.2020.06.055

**Published:** 2020-06-13

**Authors:** Leonidas Chardalias, Antonios Gklavas, Ira Sotirova, Erasmia Vlachou, John Kontis, Ioannis Papaconstantinou

**Affiliations:** a 2nd Department of Surgery, University Hospital Aretaieion, Athens, Greece; b NIMTS Army Veterans Hospital, Athens, Greece

**Keywords:** PET CT, positron emission tomography–computed tomography, HIPEC, intraperitoneal hyperthermic chemotherapy, IPAA, ileal pouch–anal anastomosis, StuDoQ Registry, Studien-, Dokumentations- und Qualitätszentrum der Deutschen Gesellschaft für Allgemein- und Viszeralchirurgie, CRC, colorectal cancer, ChT, chemotherapy, Familial adenomatous polyposis, Peritoneal carcinomatosis, Hyperthermic intraperitoneal chemotherapy, J-Pouch, Liver metastases, Case report

## Abstract

**Introduction:**

Cytoreductive surgery (CRS) with hyperthermal intraperitoneal chemotherapy (HIPEC) are established treatments for peritoneal carcinomatosis that prolong survival in carefully selected patients. At the time of diagnosis, 4–7% of patients with colorectal cancer (CRC) have metastasis to the peritoneum. There is a lack of evidence in the literature if J-pouch can be applied simultaneously with HIPEC to improve quality of life in patients with familial adenomatous polyposis syndrome (FAP) and peritoneal carcinomatosis.

**Case presentation:**

We describe a case of a 41-year-old Caucasian male with Familial Adenomatous Polyposis which was diagnosed as metastatic colorectal cancer in the liver and peritoneum. He was treated with systemic chemotherapy followed by total proctocolectomy with a J-shaped IPAA, liver metastasectomy, right hemidiaphragm resection, CRS and HIPEC.

**Discussion:**

CRS and HIPEC have been implicated with high morbidity and mortality rates. A major independent risk factor correlated with high morbidity is anastomotic failure. J-Pouch formation although considered a technique with high complication rates, improves the quality of life of patients after total proctocolectomy and is related to high patient satisfaction. There are inconclusive data on whether anastomotic failure rates are higher when performing J-Pouch and HIPEC together.

**Conclusions:**

J-Pouch after CRS and HIPEC can be offered as a treatment as long as the patient is carefully selected, in high volume centers with experienced surgeons.

## Highlights

• Peritoneal carcinomatosis with concurrent liver metastases should not be treated as end stage disease.
• IPAA anastomosis can be applied simultaneously with HIPEC, improving quality of life especially for Familial Adenomatosis Polyposis patients.
• HIPEC as a prognostic factor of anastomotic healing; ileorectal anastomosis.
• Liver metastasectomy with cytoreductive surgery and HIPEC may prolong survival.

## 1 Introduction

FAP is an autosomal dominant syndrome with an incidence of 1:10000. However, approximately 20% of cases appear as de novo mutations. The driving mutation is in the APC gene. Hereditary polyposis syndromes represent 5–10% of all CRCs. FAP usually progresses to CRC by the 4th decade of life unless prophylactic colectomy or proctocolectomy is performed [1]. Restorative proctocolectomy with ileal pouch-anal anastomosis (IPAA) is considered to be the ‘gold standard’ in the management of FAP in order to prevent cancer development and preserve fecal continence, improving quality of life.

The liver is the most common site for metastases that occur in CRC; 25–30% of patients with CRC develop liver metastatic disease [2]. The peritoneum is the second most common site of CRC metastasis (4–13%), found in 4–7% of patients at the time of diagnosis [3,4]. Treatment of peritoneal carcinomatosis from CRC is multimodal; the standard of care in carefully selected patients is CRS with HIPEC and systemic chemotherapy, the combination of which has been shown to expand the overall survival from 6 months if left untreated to 20–63 months [5]. On the other hand, CRS with HIPEC has been criticized as a surgical procedure with high morbidity and mortality rates, correlated with increased risk for anastomotic leak [6]. We present the case of an adult male with FAP which was diagnosed as metastatic CRC in the liver and peritoneum and was treated with systemic chemotherapy followed by total proctocolectomy with a J-shaped IPAA, liver metastasectomy, right hemidiaphragm resection, CRS and HIPEC. We describe the case presentation according to the SCARE guideline [7].

## 2 Case presentation

**Fig. 1:**
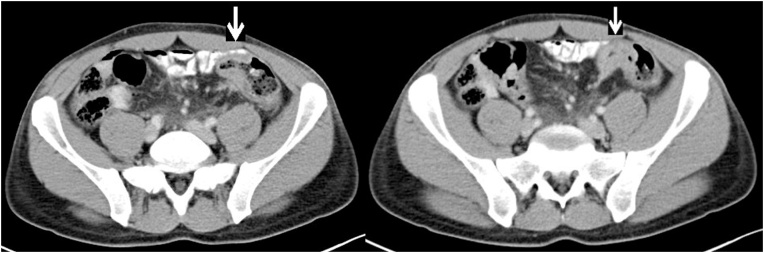
Abdominal CT revealed stenosis at the transition of the descending to sigmoid colon.

**Fig. 2:**
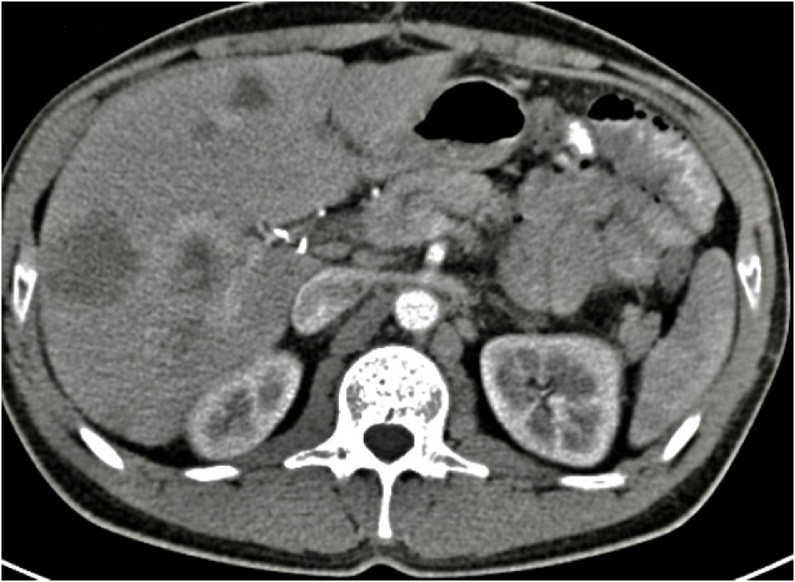
Abdominal computed tomography image demonstrating liver metastasis at the time of the diagnosis.

A 41-year-old Caucasian male with FAP and metastatic colon cancer was referred to our surgical oncology department, at an academic institution. His weight was 65 kg (BMI 22.5), he had no other comorbidities and was working as a construction worker. The patient had a family history of FAP, with his mother and sister dying of FAP related colon cancer at the age of 32 and 34 respectively. Despite that, he refused the recommended screening program. Two years ago he presented to a private hospital with atypical epigastric symptoms and change in bowel habits over the past month. Colonoscopy, at that time, revealed multiple polyps of the rectum, sigmoid and distal descending colon; a total colonoscopy was not achieved due to the neoplastic stenosis of the descending colon from which biopsies were obtained. Upper gastrointestinal tract endoscopy showed multiple cystic polyps at the gastric fundus and body and multiple duodenal adenomas at the ampulla of Vater. Abdominal Computerized Tomography (CT) revealed stenosis at the transition of the descending to the sigmoid colon, multifocal metastatic disease of the liver and a mesenteric nodal mass (1.9 cm) (Fig. 1, Fig. 2). Meanwhile, the patient developed obstructive symptoms within a few days and therefore an exploratory laparotomy was performed, in which diffuse peritoneal carcinomatosis was revealed in combination with an obstructing neoplastic mass in the aforementioned, by the colonoscopy and CT, segment of descending colon. A diverting loop ileostomy was created, 20 cm from ileocecal junction and biopsies were obtained from the liver and the greater omentum.

Histopathological examination of the tissue obtained by colonoscopy and surgical specimens revealed moderately differentiated adenocarcinoma. Immunohistochemistry tests were positive for the markers CDX2 and CK20, whereas the CK7 marker was negative. KRAS/BRAF testing of pathological specimens revealed wild-type KRAS, wild-type NRAS, non-mutant BRAF gene. and a microsatellite stable (MSS) tumor.

Afterward, he was referred to our department. The case was discussed in our institution’s cancer multidisciplinary team (MDT), which consists of clinical oncologists, surgeons, radiologists, pathologists and radiation oncologists. Chemotherapy (ChT) was decided for 15 months, consisting of Oxaliplatin and Bevacizumab regimens.

## 3 Clinical findings

On physical examination, he appeared well-nourished, afebrile and in good general condition. The abdominal examination revealed no palpable masses or other findings, and the presence of a loop ileostomy on the right lower quadrant.

## 4 Timeline

**Table:** 

Date	Information
**September 2017**	Atypical epigastric symptoms, change in bowel habits. Family history of FAP. Colonoscopy: multiple polyps of the rectum, sigmoid and distal descending colon; a total colonoscopy was not achieved due to the neoplastic stenosis of the descending colon. Upper GI tract endoscopy showed multiple cystic polyps at the gastric fundus and body and 3 duodenal adenomas at the ampulla of Vater CT showed stenosis at the transition of the descending to the sigmoid colon, multifocal metastatic disease. Obstructive symptoms Underwent exploratory laparotomy: diffuse carcinomatosis, biopsies, loop ileostomy creation at a private hospital. Pathology Anatomy result: moderately differentiated adenocarcinoma.
**November 2017-February 2019**	Referred to our surgical oncology department MDT recommendation: ChT with Oxaliplatin and Bevacizumab
**February 2019**	CT and MRI revealed an impressive response of metastatic liver disease; only a single metastatic lesion in liver segment VI was detected, which had been downsized from 3.5 cm before ChT to 1.8 cm after ChT. PET-CT revealed only one hypermetabolic area (SUVmax = 4.2) in liver segment VI.
**May 2019**	He underwent loop ileostomy resection, total proctocolectomy with J-Pouch formation with diverting ileostomy, liver metastasectomy, part of right hemidiaphragm resection and HIPEC
**August 2019–December 2019**	ChT with bevacizumab and capecitabine regimens
**December 2019**	Upper GI endoscopy: no polyps found in stomach, or duodenum Normal pouchogram
**February 2020**	Ileostomy reversal Scheduled for PET CT and re-evaluation by the MDT afterward

## 5 Diagnostic assessment

Full blood count and biochemical test were within normal limits (WBC: 5.000/uL, PLT: 229,000/uL, CEA: 3.84 ng/mL, Alb: 4.9 g/dL, SGOT: 19U/L, SGPT:14 U/L, ALP: 84 U/L, γ-GT: 26 U/L, T.bili: 0.35 mg/dL, Urea: 40.2 mg/dl, Creatinine: 0.8 mg/dL)

**Fig. 3:**
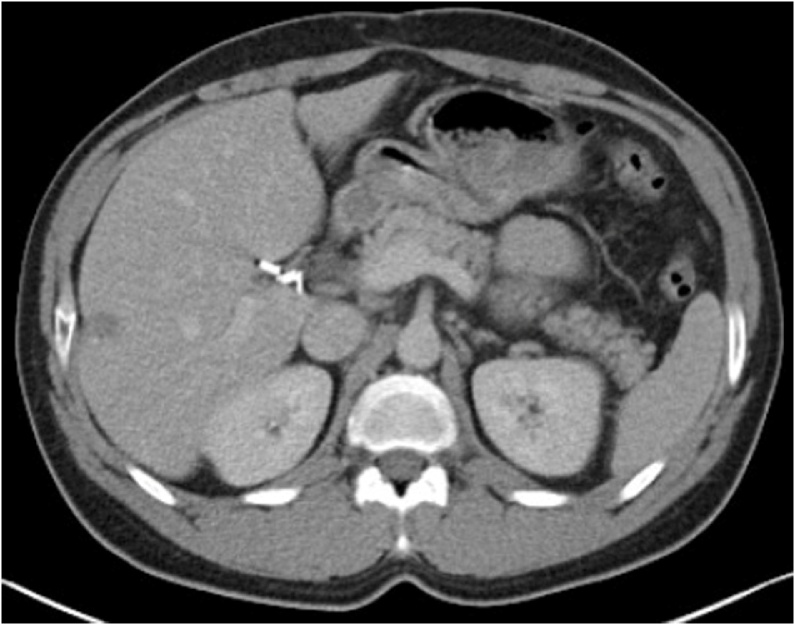
Abdominal computed tomography image demonstrating liver metastasis after chemotherapy.

**Fig. 4:**
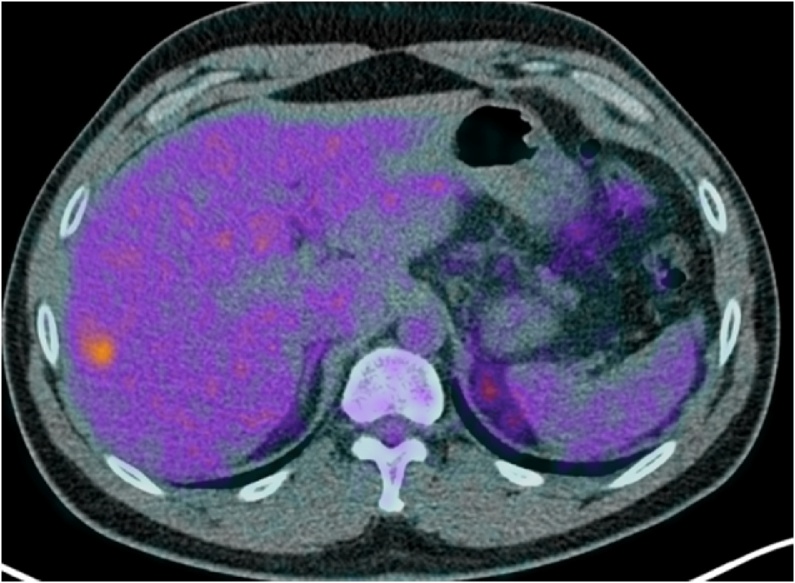
Positron emission tomography–computed tomography after chemotherapy revealed only one hypermetabolic area (SUVmax = 4.2) in liver segment VI.

Subsequent CT and Magnetic Resonance Imaging (MRI) revealed an impressive response of metastatic liver disease; only a single metastatic lesion in liver segment VI was detected, which had been downsized from 3.5 cm before ChT to 1.8 cm after ChT. Moreover, multiple small focal lesions were shown in liver segments III and VI, possibly representing “cured” metastases (Fig. 3). Positron emission tomography–computed tomography of abdomen-thorax and brain (PET-CT) revealed only one hypermetabolic area (SUVmax = 4.2) in liver segment VI. Interestingly, PET-CT showed absence of hypermetabolic neoplastic activity elsewhere in the abdominal cavity, including the already known site of the primary tumor in the descending colon (Fig. 4).

## 6 Therapeutic intervention

Our hospital’s multidisciplinary tumor board recommended that a second exploratory laparotomy in combination with possible restorative proctocolectomy, liver metastasectomy, peritonectomy and HIPEC should be offered to the patient.

Abdominal cavity exploration with intraoperative ultrasonography (IOUS) revealed seven liver metastases (segment II and right lobe), metastatic lesions to the right hemidiaphragm and three suspicious lesions in the mesentery. Peritoneal carcinomatosis Index (PCI) was 7.

He then underwent loop ileostomy resection, total proctocolectomy with J-Pouch formation and diverting ileostomy, liver metastasectomy, partial resection of right hemidiaphragm and HIPEC. The IOUS was reperformed to rule out any remaining liver metastases.

The small bowel mesentery was mobilized adequately, the previous loop ileostomy was transected with a 75 mm linear stapler and J-Pouch was constructed from the distal 40 cm of ileum with multiple firings of the 75 mm linear stapler via enterotomy at the pouch apex. The blind loop of the J-pouch was reinforced with an overstitch line. Insufflation with normal saline confirmed the integrity of the pouch. The IPAA anastomosis was performed with a circular stapler. Care was taken for the prevention of twisting of small bowel mesentery. The doughnuts were checked and the air test was negative for leakage from the suture lines. Last, a diverting ileostomy was performed.

Completeness of cytoreduction (CCR) score was 0. The open coliseum technique was utilized for HIPEC, suspending the edges of the abdominal wall with a running suture over a self-retaining retractor. HIPEC with mitomycin C was administered for 90 min, at a temperature of 42 °C simultaneously with intravenous administration of 5-Fluorouracil and leucovorin. After HIPEC had been completed, the abdomen was washed out.

The postoperative course was complicated with persistent fever at day 7 (Clavien-Dindo Classification IIIa). Proper workup was done with daily laboratory tests, blood and urine cultures, chest x-ray and empiric antibiotic therapy. *Escherichia coli* was isolated from the intra-port blood culture and targeted antibiotic therapy was given. Intra-port removal was performed and the fever resolved a few days after. This complication prolonged his hospital stay to 15 days.

## 7 Follow-up and outcomes

**Fig. 5:**
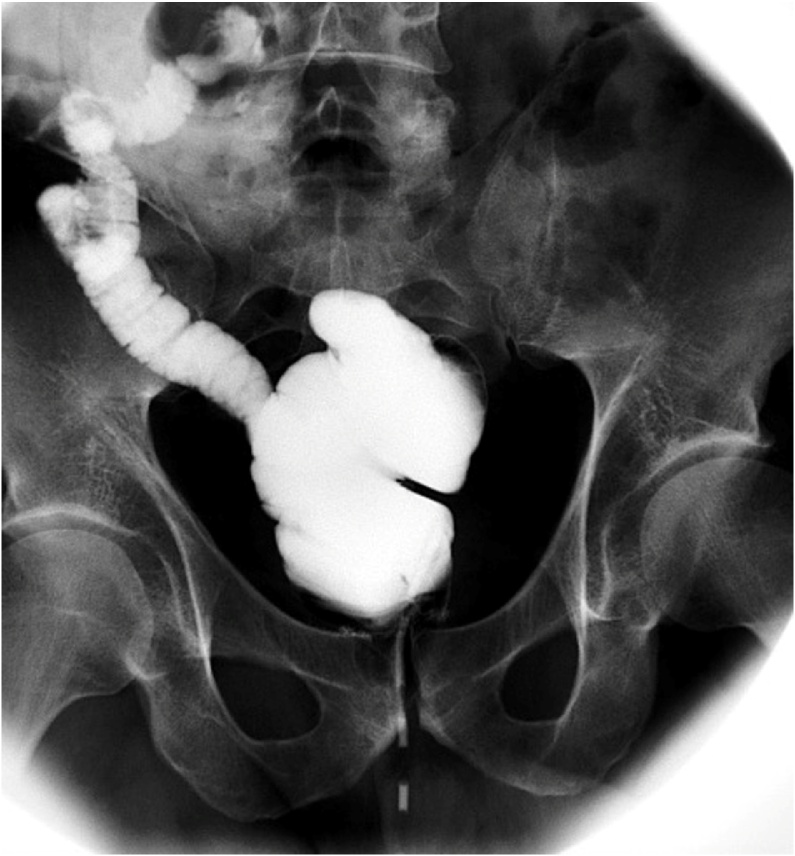
A pouchogram of the patient 6 months after ileal pouch-anal anastomosis performed before diverting stoma closure.

Subsequently, the patient underwent ChT for six months with bevacizumab and capecitabine regimens according to our tumor MDT recommendation. Abdomen and thorax CT and MRI showed no metastasis after the chemotherapy course had been completed. Interestingly, upper GI endoscopy after ChT revealed no polyps in the stomach or duodenum. The pouchogram before the ileostomy closure was normal (Fig. 5) and the patient underwent uncomplicated ileostomy reversal. 9 months after CRS and HIPEC the patient is disease-free and scheduled for PET-CT to re-evaluate the therapeutic plan. Concerning the quality of life, the patient refers no sexual dysfunction, he is back at work, he has 3–5 bowel movements per day. According to Wexner score he has no incontinence (score 0) [8].

## 8 Discussion

FAP is an autosomal dominant inherited syndrome characterized by multiple large bowel adenomas being formed in early childhood, progressing to adenocarcinoma usually by the 4th decade of life if not treated. According to the number of colorectal polyps, FAP is categorized as classical (>100 polyps) or attenuated (10–100 polyps) [9]. Approximately 20% of patients have de novo mutations; due to the lack of family history these patients seek medical consultation only when the disease becomes symptomatic (polyps, CRC) [10].

Our patient had shown poor compliance in medical consultation and was not under screening, although he had a family history of FAP. As a result, he presented with CRC that caused bowel obstruction with concomitant liver and peritoneal metastases.

Peritoneal carcinomatosis standard therapy for certain types of cancer (e.g CRC) has been established as complete CRS with HIPEC [4]. Some theories have been proposed regarding the possible negative effect of CRS and HIPEC in the process of anastomotic healing; intestinal edema caused by this longstanding surgical procedure, the need for excessive fluid resuscitation, as long as the thermal pressure and the cytotoxicity of the regimens used during HIPEC have been considered as aggravating factors in the process of anastomotic healing [11,12].

CRS and HIPEC have been implicated with high morbidity and mortality rates. A major independent risk factor correlated with high morbidity is anastomotic failure. Anastomotic leakage rates range from 0 to 9% in experienced centers [6]. The German DGAV StuDoQ Registry(Studien-, Dokumentations- und Qualitätszentrum der Deutschen Gesellschaft für Allgemein- und Viszeralchirurgie), the largest published national registry on CRS and HIPEC with 2149 patients, reports anastomotic leakage rates of 6% with rectal anastomosis being an independent prognostic factor [13]. Anastomotic failure seems to be dependent the on patient’s gender or tumor histology but not on anastomotic technique [14]. However, Paul Sugarbaker, who first developed CRS and HIPEC (Sugarbaker procedure) published a series of 29 patients without an anastomotic leak. This was attributed to a second, reinforcing overstitch line in the stapled colorectal anastomosis [15].

IPAA after total proctocolectomy is well-established as it improves the quality of life of patients and is related to high patient satisfaction and good or excellent functional outcomes for up to 95% of patients [16]. Nevertheless, its complications-acute postoperatively or late-range from 30 to 60% among studies; they include septic complications such as anastomotic leak, fistula or pouchitis and non-septic ones such as small bowel obstruction, stricture, pouchitis and cuffitis which can lead to pouch failure [17]. Mortality rates range from 3.5–17% [18]. Anastomotic leak, the most feared complication, occurs in 9% of patients with FAP and J-Pouch [19]. Inflammatory bowel disease, body mass index greater than 30, patient older than 50 years and surgeon’s inexperience have been presented as risk factors for pouch complications [20]. Searching the literature, we found a case series of 5 patients who were treated with simultaneous J pouch formation and HIPEC, with no complications reported in their postoperative period [21].

This is an interesting case of simultaneous liver metastasectomy, CRS, restorative proctocolectomy with J-Pouch formation and HIPEC. The patient is free of metastasis according to postoperative follow-up with an uncomplicated functional pouch and a reversed ileostomy 9 months after surgery.

It seems that J-Pouch after CRS and HIPEC can be offered as a treatment as long as the patient is carefully selected, in high-volume centers with experienced surgeons. However, this is a single case report and this kind of treatment cannot be established unless more patients are treated in designated tertiary referral centers with positive outcome, in order to offer patients not only a prolonged overall survival but an acceptable quality of life, as well.

## Declaration of Competing Interest

None.

## Funding

None.

## Ethical approval

Ethical approval is not required for case reports in my institution.

## Consent

Written informed consent was obtained from the patient for publication of this case report and any accompanying images. A copy of the written consent is available for review by the Editor-in-Chief of this journal.

## Registration of research studies

NA.

## Guarantor

Ioannis Papakonstantinou.

Leonidas Chardalias.

## Provenance and peer review

Not commissioned, externally peer-reviewed.

## CRediT authorship contribution statement

**Leonidas Chardalias:** Writing - original draft, Writing - review & editing, Visualization. **Antonios Gklavas:** Writing - review & editing. **Ira Sotirova:** Visualization. **Erasmia Vlachou:** Resources. **John Kontis:** Supervision. **Ioannis Papaconstantinou:** Supervision.
